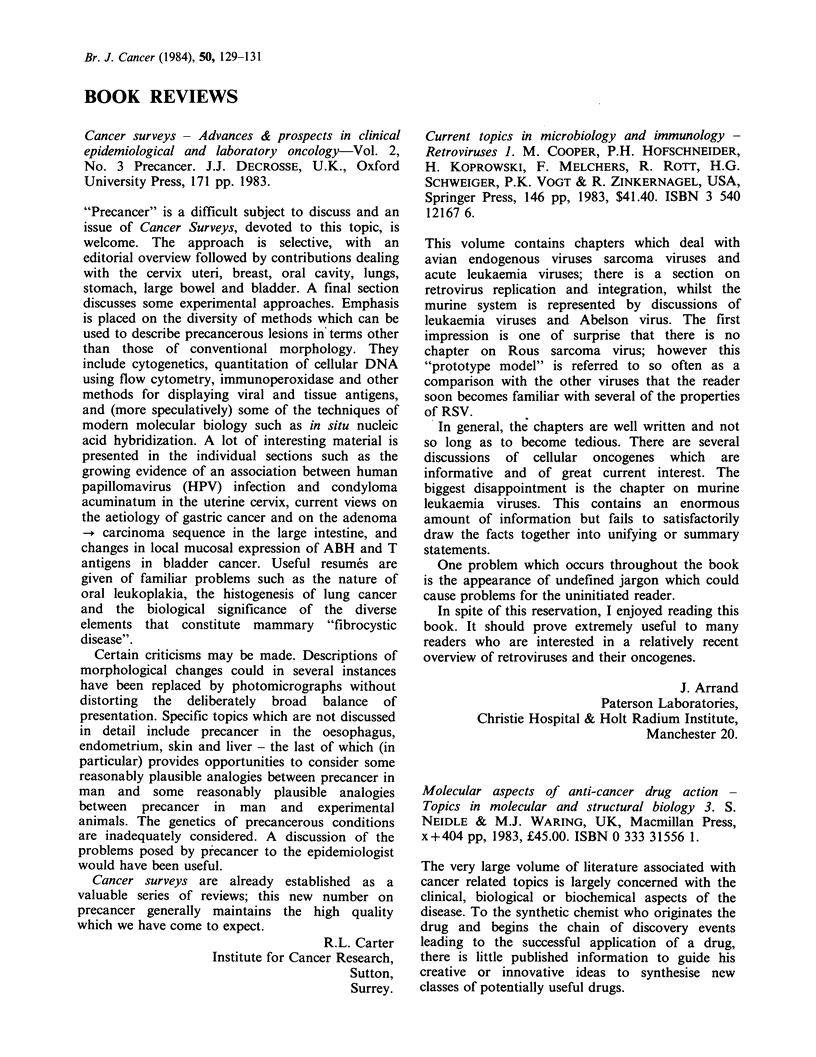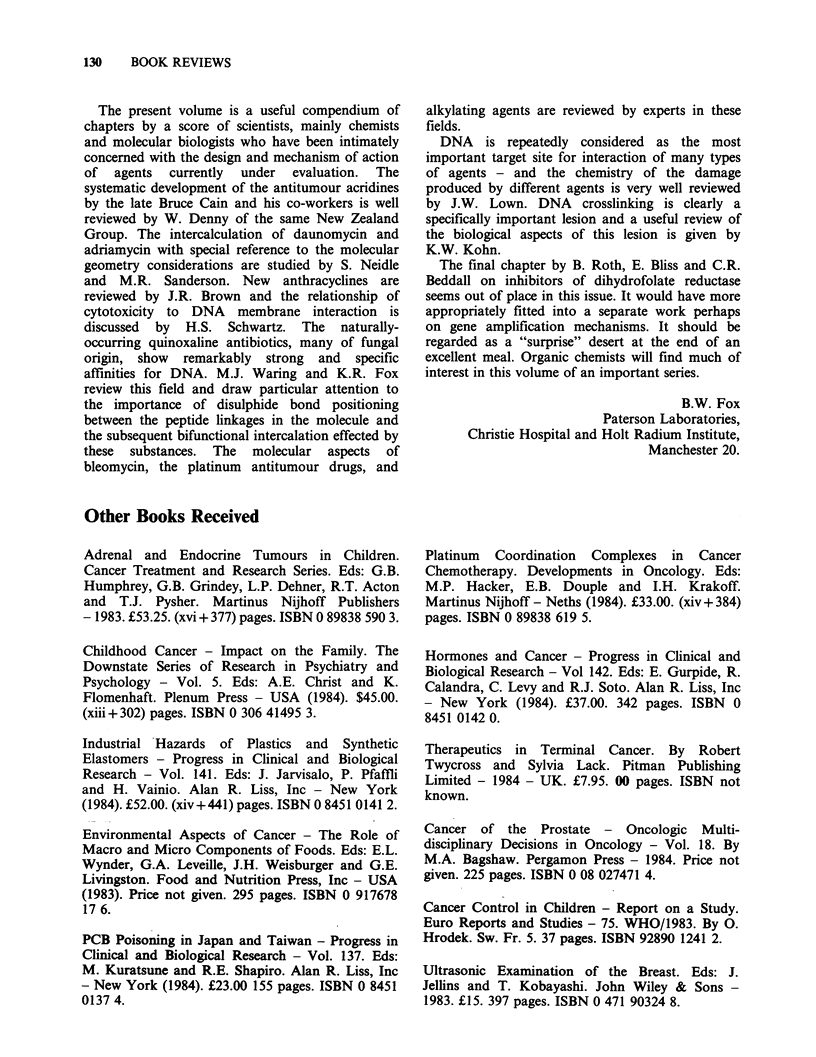# Molecular aspects of anti-cancer drug action Topics in molecular and structural biology

**Published:** 1984-07

**Authors:** B.W. Fox


Paterson Laboratories,

Christie Hospital & Holt Radium Institute,

Manchester 20.

*Molecular aspects of anti-cancer drug action – Topics in molecular and structural biology 3.* S. NEIDLE & M.J. WARING, UK, Macmillan Press, x+404 pp, 1983, £45.00. ISBN 0 333 31556 1.

The very large volume of literature associated with cancer related topics is largely concerned with the clinical, biological or biochemical aspects of the disease. To the synthetic chemist who originates the drug and begins the chain of discovery events leading to the successful application of a drug, there is little published information to guide his creative or innovative ideas to synthesise new classes of potentially useful drugs.

The present volume is a useful compendium of chapters by a score of scientists, mainly chemists and molecular biologists who have been intimately concerned with the design and mechanism of action of agents currently under evaluation. The systematic development of the antitumour acridines by the late Bruce Cain and his co-workers is well reviewed by W. Denny of the same New Zealand Group. The intercalculation of daunomycin and adriamycin with special reference to the molecular geometry considerations are studied by S. Neidle and M.R. Sanderson. New anthracyclines are reviewed by J.R. Brown and the relationship of cytotoxicity to DNA membrane interaction is discussed by H.S. Schwartz. The naturally-occurring quinoxaline antibiotics, many of fungal origin, show remarkably strong and specific affinities for DNA. M.J. Waring and K.R. Fox review this field and draw particular attention to the importance of disulphide bond positioning between the peptide linkages in the molecule and the subsequent bifunctional intercalation effected by these substances. The molecular aspects of bleomycin, the platinum antitumour drugs, and alkylating agents are reviewed by experts in these fields.

DNA is repeatedly considered as the most important target site for interaction of many types of agents – and the chemistry of the damage produced by different agents is very well reviewed by J.W. Lown. DNA crosslinking is clearly a specifically important lesion and a useful review of the biological aspects of this lesion is given by K.W. Kohn.

The final chapter by B. Roth, E. Bliss and C.R. Beddall on inhibitors of dihydrofolate reductase seems out of place in this issue. It would have more appropriately fitted into a separate work perhaps on gene amplification mechanisms. It should be regarded as a "surprise" desert at the end of an excellent meal. Organic chemists will find much of interest in this volume of an important series.

B.W. Fox
Paterson Laboratories,
Christie Hospital and Holt Radium Institute,
Manchester 20.